# Malleting

**Published:** 1875-05

**Authors:** H. V. Kagey

**Affiliations:** Dentist, Arcola, Ill.


### MALLETING.

BY H. V. KAGEY, DENTIST, ARCOLA, ILL.

"Be not the first, by whom the new is tried;
Not the last, to lay the old aside."

"Over malleting is one of the evils of the practice of the day," Dental Cosmos, No.1 vol. xvii, page 54. Here is the ring of a characteristic tone, which seems to be cropping out, more or less, in all our recent conventions. We welcome it as the harbinger of more matured and conservative thought. It wins the appellation of heroic—when weak, timorous humanity, can stand up on her own individuality and declare her conscientious scruples! What craven, tongue-tied slaves we are to public opinion. As a lamb before the shearer, so are we before the popular party. Four years ago, had Dr. Mowbray made his statement, he would not have subjected his gold pellets to severe malleting, but he would have subjected his golden reputation to the loud sounding knocks of anathematized plugger—the tidal party. Further, he says, "no more two hundred and seventy or three hundred and eighty blows of mallet on a pellet of gold in my practice—believe one hundred blows will greatly injure if not altogether destroy its cohesiveness." Now, it is my opinion that even fifty strokes on a medium pellet will greatly impair its molecular affection.

Three hundred and eighty! Ye gods! With an automatic alone, will knock in the feathers of the American bird on a gold coin, and leave him shapeless as the man in the moon. We may pound gold till our hair is silvered over, we cannot make the density greater than cast gold. But we can, in less time than it takes to tell it, produce such a molecular effect by our constant, "knocking knocking" as to leave it stiff and rigid. With large sized foil tweezers take up a pellet—I care not the No. from 6 to 240—even a piece of soft plate,—and observe what little force is required to squeeze its sides into perfect contact. After this perfect contact has been made, all further pressure in the same direction is simply to change its form, but not its density. How often on the page of dental literature has this would be fact of importance stared us in the face?

"No filling can be perfectly condensed by hand pressure, etc." Now I don't say this enunciation is a lie; I think it's simply a fungus, attending those hot spurred efforts to float on the wave of the eccentrical current—"only that, and nothing more." So far as the required density is concerned, any gold filling can be amply accomplished by hand pressure. But in very many cases I prefer the auto-mallet, because it is easier on me; and for the same reason, I know good operaters, who prefer the steel and lead mallet. My lead mallet has often led to golden results; but now lies down with its laurel crown, and my feelings are," disturb him not, but let him rest." Good operators use it, and good operations are performed with it; but why use that which necessitates the presence of a second party when a little silver dressed automatic mallet will answer in place? When the epidemic of heavy foils and heavy mallets raged I was told by a friend, a gentleman and a scientific operator, that unless I used these articles, my operations could never compete with perfect work. Each year I find myself gravitating nearer the conviction, that he was mistaken. Some times, I am inclined to think there would have been more truth if the oppesite had been spoken.

Now I put it to you my respected co-laborers for good, have your observatious not been as mine? I have found in the enamel around many beautiful fillings made with the mallet, fractures which, I am pained to say, were the result of malleting. These observations were from my own work, as well, as from that of others. (I, thus,court the good of my soul by free confession.) Nor do I believe this was always done by "over malleting." We all agree that a good filling must necessarily be up close against the walls. If the depth of all cavities proceeded at right angles to the orifice, and the walls of the same were parallel, we might never endanger tooth substance by mallet strokes; but we don't find cavities this way often; and, it is here, I apprehend, where the mischief is. In our laudable efforts to reach the varied concave walls in building up close, the point strikes against the wall and a fracture is the result.

But says one,"you should not let the point touch the tooth wall when it receives the mallet stroke." A reply is echoed from my pen—in many cavities of a complex, concave, posterior decay, you cannot help it. But in like cavities; without the mallet, you can, gently, let the point slide down against the wall till it reaches the gold and then give it a force amply sufficient for perfect union, and at the same time far less concentrated for fracture.

A mason resting his chisel against a nodule of marble, with all his available force, cannot effect it in the least; but a single tap from his mallett makes the calcareous child skip with a song akin to,"dont forget me mother". And it is the same principle that breaks up that once happy union in our own marble. walls

Many teeth have been well filled—have even escaped fracture, but have had their socket membranes so seriously irritated by those thumps and bumps, as never to entirely recover.

It is true, they would herald to the world we are the "elect," the golden medals about our necks are a guarantee to the high standing in the circle of our peers! Many brilliant encomiums have been pronounced over us, etc., etc.! But, alas! Science speaks; her testimony strips them of their aristocratic robe and proves the blood impure! suppuration at the root! (And just so you will often find it.) I have many fillings in my own teeth some of heavy foil and from heavy mallets and this, together with my observations of, and testimony from others, compels me to enter my protest against this over Vulcan-like pounding of the day.

